# Supplementary material for: Lifespan variation among people with a given disease or condition
Source: PLoS One. 2023 Sep 1;18(9):e0290962. doi: 10.1371/journal.pone.0290962 (PMC10473533; doi:10.1371/journal.pone.0290962)
Supplement: S1 Table — (PDF) [file pone.0290962.s001.pdf]

S1 Table. Life table for women diagnosed with mental disorders based on observed mortality rates

age	lx	dx	Lx	Tx	ex	ed
15	99941	26	99928	5776398	57.8	10.9
16	99915	17	99906	5676471	56.8	10.9
17	99898	27	99884	5576564	55.8	10.9
18	99871	41	99850	5476680	54.8	10.9
19	99829	48	99805	5376830	53.9	10.8
20	99781	59	99752	5277025	52.9	10.8
21	99723	60	99693	5177273	51.9	10.8
22	99663	62	99632	5077580	50.9	10.8
23	99601	66	99568	4977948	50	10.8
24	99536	55	99508	4878380	49	10.7
25	99480	46	99457	4778872	48	10.7
26	99434	65	99402	4679415	47.1	10.7
27	99370	56	99342	4580013	46.1	10.7
28	99314	83	99272	4480671	45.1	10.6
29	99231	88	99186	4381399	44.2	10.6
30	99142	85	99100	4282212	43.2	10.6
31	99057	112	99001	4183113	42.2	10.6
32	98945	106	98892	4084111	41.3	10.5
33	98839	106	98786	3985219	40.3	10.5
34	98732	116	98674	3886434	39.4	10.5
35	98616	119	98557	3787759	38.4	10.4
36	98498	144	98426	3689202	37.5	10.4
37	98354	143	98282	3590777	36.5	10.4
38	98211	187	98117	3492494	35.6	10.3
39	98024	155	97946	3394377	34.6	10.3
40	97869	195	97771	3296431	33.7	10.2
41	97673	238	97554	3198660	32.7	10.2
42	97435	241	97314	3101106	31.8	10.1
43	97194	265	97061	3003791	30.9	10.1
44	96929	355	96752	2906730	30	10
45	96574	342	96404	2809978	29.1	10
46	96233	387	96039	2713575	28.2	9.9
47	95846	483	95604	2617535	27.3	9.8
48	95362	581	95072	2521931	26.4	9.7
49	94782	611	94476	2426860	25.6	9.6
50	94171	628	93857	2332383	24.8	9.5
51	93543	761	93163	2238526	23.9	9.4
52	92782	854	92356	2145363	23.1	9.3
53	91929	915	91471	2053008	22.3	9.2
54	91014	925	90551	1961536	21.6	9.1
55	90089	1155	89511	1870985	20.8	8.9
56	88933	1238	88314	1781474	20	8.8

57	87695	1250	87070	1693159	19.3	8.6
58	86445	1367	85761	1606089	18.6	8.5
59	85078	1458	84349	1520328	17.9	8.3
60	83620	1596	82822	1435979	17.2	8.2
61	82024	1775	81136	1353157	16.5	8
62	80249	1756	79371	1272021	15.9	7.8
63	78493	2021	77483	1192650	15.2	7.6
64	76472	2043	75450	1115167	14.6	7.5
65	74428	2224	73317	1039717	14	7.3
66	72205	2161	71124	966401	13.4	7.1
67	70043	2252	68917	895277	12.8	6.9
68	67791	2396	66593	826360	12.2	6.7
69	65394	2371	64209	759767	11.6	6.5
70	63024	2533	61757	695558	11	6.3
71	60491	2707	59138	633801	10.5	6.1
72	57784	2913	56328	574663	9.9	5.9
73	54871	3189	53277	518335	9.4	5.7
74	51683	3009	50178	465058	9	5.5
75	48673	3196	47075	414880	8.5	5.3
76	45477	3135	43910	367805	8.1	5.1
77	42342	3065	40810	323895	7.6	4.9
78	39277	3214	37670	283086	7.2	4.7
79	36064	3125	34501	245415	6.8	4.5
80	32938	3184	31347	210914	6.4	4.3
81	29755	2887	28311	179568	6	4.1
82	26868	3059	25338	151256	5.6	3.9
83	23809	2891	22363	125918	5.3	3.7
84	20918	2801	19517	103555	5	3.6
85	18116	2570	16831	84038	4.6	3.4
86	15546	2303	14395	67207	4.3	3.2
87	13244	2212	12137	52812	4	3
88	11031	2085	9989	40674	3.7	2.8
89	8946	1813	8040	30686	3.4	2.7
90	7133	1555	6356	22646	3.2	2.5
91	5578	1382	4887	16290	2.9	2.4
92	4196	1084	3654	11403	2.7	2.2
93	3112	912	2656	7749	2.5	2.1
94	2200	702	1849	5093	2.3	2
95	1498	504	1246	3244	2.2	1.9
96	994	357	815	1998	2	1.7
97	637	241	516	1183	1.9	1.6
98	395	172	309	667	1.7	1.6
99	223	101	173	358	1.6	1.5
100	122	57	94	185	1.5	1.4

Source: Authors' calculations based on mortality data from Danish Registers

Note. l_x = survivors to age x, d_x = deaths at age x, L_x = person-years lived at age x, T_x = person-years lived above age x, e_x = life expectancy at age x, ed = life disparity at age x. Results in this study were calculated based on mortality data from 0 to 99+ years, but results are not presented for ages below 15 years due to a low number of individuals.
