# Supplementary material for: Lifespan variation among people with a given disease or condition
Source: PLoS One. 2023 Sep 1;18(9):e0290962. doi: 10.1371/journal.pone.0290962 (PMC10473533; doi:10.1371/journal.pone.0290962)
Supplement: S2 Table — (PDF) [file pone.0290962.s002.pdf]

S2 Table. Life table for women from the general population

age	lx	dx	Lx	Tx	ex	ed
15	99,490	22	99,479	6,580,836	66.1	10
16	99,468	25	99,455	6,481,357	65.2	10
17	99,443	27	99,430	6,381,902	64.2	10
18	99,416	21	99,406	6,282,472	63.2	10
19	99,395	14	99,388	6,183,066	62.2	10
20	99,381	14	99,374	6,083,678	61.2	10
21	99,367	19	99,358	5,984,303	60.2	10
22	99,349	22	99,338	5,884,945	59.2	9.9
23	99,327	18	99,318	5,785,608	58.2	9.9
24	99,309	25	99,296	5,686,290	57.3	9.9
25	99,284	30	99,269	5,586,994	56.3	9.9
26	99,254	23	99,243	5,487,724	55.3	9.9
27	99,231	25	99,219	5,388,482	54.3	9.9
28	99,207	32	99,191	5,289,263	53.3	9.9
29	99,175	35	99,158	5,190,072	52.3	9.9
30	99,140	30	99,125	5,090,914	51.4	9.9
31	99,110	30	99,096	4,991,789	50.4	9.8
32	99,081	36	99,063	4,892,693	49.4	9.8
33	99,045	38	99,026	4,793,631	48.4	9.8
34	99,007	49	98,983	4,694,604	47.4	9.8
35	98,958	55	98,930	4,595,622	46.4	9.8
36	98,903	53	98,876	4,496,691	45.5	9.8
37	98,849	59	98,819	4,397,816	44.5	9.7
38	98,790	73	98,753	4,298,996	43.5	9.7
39	98,717	77	98,678	4,200,243	42.5	9.7
40	98,640	84	98,598	4,101,565	41.6	9.7
41	98,556	100	98,506	4,002,967	40.6	9.6
42	98,456	117	98,397	3,904,461	39.7	9.6
43	98,338	133	98,272	3,806,064	38.7	9.6
44	98,206	158	98,127	3,707,792	37.8	9.5
45	98,048	168	97,964	3,609,665	36.8	9.5
46	97,879	177	97,791	3,511,702	35.9	9.4
47	97,702	215	97,595	3,413,911	34.9	9.4
48	97,488	233	97,371	3,316,316	34	9.3
49	97,255	239	97,135	3,218,945	33.1	9.3
50	97,016	269	96,881	3,121,809	32.2	9.2
51	96,747	345	96,574	3,024,928	31.3	9.2
52	96,402	376	96,214	2,928,354	30.4	9.1
53	96,026	369	95,841	2,832,140	29.5	9

54	95,657	403	95,455	2,736,299	28.6	8.9
55	95,254	453	95,027	2,640,844	27.7	8.8
56	94,800	496	94,552	2,545,817	26.9	8.8
57	94,304	533	94,038	2,451,265	26	8.7
58	93,771	571	93,485	2,357,227	25.1	8.6
59	93,200	581	92,909	2,263,742	24.3	8.5
60	92,619	646	92,296	2,170,833	23.4	8.4
61	91,973	758	91,594	2,078,537	22.6	8.3
62	91,215	792	90,819	1,986,943	21.8	8.2
63	90,423	818	90,014	1,896,124	21	8
64	89,604	895	89,157	1,806,111	20.2	7.9
65	88,709	978	88,220	1,716,954	19.4	7.8
66	87,731	1,058	87,202	1,628,734	18.6	7.7
67	86,673	1,114	86,116	1,541,532	17.8	7.6
68	85,559	1,227	84,945	1,455,416	17	7.4
69	84,331	1,379	83,642	1,370,471	16.3	7.3
70	82,952	1,555	82,175	1,286,829	15.5	7.2
71	81,397	1,689	80,553	1,204,654	14.8	7
72	79,708	1,812	78,802	1,124,102	14.1	6.8
73	77,896	1,969	76,911	1,045,300	13.4	6.7
74	75,927	2,121	74,866	968,388	12.8	6.5
75	73,806	2,287	72,662	893,522	12.1	6.3
76	71,518	2,468	70,285	820,860	11.5	6.2
77	69,051	2,573	67,764	750,575	10.9	6
78	66,478	2,709	65,123	682,811	10.3	5.8
79	63,769	2,918	62,310	617,688	9.7	5.6
80	60,851	3,074	59,314	555,378	9.1	5.5
81	57,776	3,262	56,145	496,065	8.6	5.3
82	54,514	3,440	52,794	439,919	8.1	5.1
83	51,074	3,501	49,324	387,125	7.6	4.9
84	47,573	3,584	45,781	337,802	7.1	4.7
85	43,989	3,751	42,113	292,021	6.6	4.6
86	40,238	3,818	38,328	249,907	6.2	4.4
87	36,419	3,793	34,523	211,579	5.8	4.2
88	32,626	3,799	30,726	177,056	5.4	4
89	28,827	3,734	26,960	146,330	5.1	3.9
90	25,093	3,544	23,321	119,370	4.8	3.7
91	21,549	3,328	19,884	96,049	4.5	3.6
92	18,220	3,085	16,678	76,165	4.2	3.5
93	15,135	2,779	13,745	59,487	3.9	3.3
94	12,356	2,429	11,141	45,741	3.7	3.2

95	9,927	2,148	8,853	34,600	3.5	3.1
96	7,779	1,802	6,878	25,747	3.3	3.1
97	5,977	1,480	5,237	18,869	3.2	3
98	4,497	1,203	3,896	13,632	3	3
99+	3,294	3,294	9,736	9,736	3	3

Source: Authors' calculations based on mortality data from Statistic Denmark.

Note. l_x = survivors to age x, d_x = deaths at age x, L_x = person-years lived at age x, T_x = person-years lived above age x, e_x = life expectancy at age x, ed = life disparity at age x. Results in this study were calculated based on mortality data from 0 to 99+ years, but results are not presented for ages below 15 years, so as to be consistent with the results for women diagnosed with any mental disorder.
