# Supplementary material for: Lifespan variation among people with a given disease or condition
Source: PLoS One. 2023 Sep 1;18(9):e0290962. doi: 10.1371/journal.pone.0290962 (PMC10473533; doi:10.1371/journal.pone.0290962)
Supplement: S1 File — (PDF) [file pone.0290962.s003.pdf]

```
library(DemoDecomp)
### functions
# calculating a0, i.e., ax at age 0
a0 <- function(m0, sex){
  if (sex=="F"){
    if(m0<0.01724){a0<-0.14903-2.05527*m0}
    if(m0>=0.01724&m0<0.06891){a0 <- 0.04667+3.88089*m0}
    if(m0>=0.06891){a0 <- 0.31411}
  }
  if (sex=="M"){
    if(m0<0.02300){a0<-0.14929-1.99545*m0}
    if(m0>=0.02300&m0<0.08307){a0 <- 0.02832+3.26021*m0}
    if(m0>=0.08307){a0 <- 0.29915}
  }
  return(a0)}

# obtaining different metrics, namely, life expectancy (ex), life disparity (ed),
lifetable entropy (Hx)
# age = different ages, i.e., 0, 1, 2, 3,…, mx = rates of mortality, wx = weight of the
calculation, i.e., the number of people diagnosed with mental disorders at each age of
onset, sex = men/women are parameters for the calculation
Metrics <- function(age, mx, wx, sex){
  m <- length(age)
  OPENAGE <- m - 1
  RADIX <- 1
  ax <- ifelse(mx==0, 0, mx*0+0.5)
  ax[1] <- a0(mx[1], sex[1])
  qx <- mx/(1+(1-ax)*mx)
  qx[m] <- ifelse(is.na(qx[m]), NA, 1)
  ax[m] <- ifelse(mx[m]==0, 0, 1/mx[m])
  px <- 1 - qx
  px[is.nan(px)] <- 0
  lx <- c(RADIX, RADIX*cumprod(px[1:OPENAGE]))
  dx <- lx*qx
  Lx <- lx-(1-ax)*dx
  Lx[m] <- lx[m]*ax[m]
  Tx <- rev(cumsum(rev(Lx)))
  ex <- Tx/lx
  dx.lx <- c(dx[-m]*(ex[-m]+ax[-m]*(ex[-1]-ex[-m])), dx[m]*ex[m])
  ed <- rev(cumsum(rev(dx.lx)))/lx
  Hx <- ed/ex
  wx <- wx
  Results <- data.frame(age, ex, ed, Hx, wx)
  return(Results)}

# calculating the average remaining life expectancy after disease diagnosis
avgex <- function(mx, wx, sex){
  m <- length(mx)
  OPENAGE <- m - 1
  RADIX <- 1
  ax <- ifelse(mx==0, 0, mx*0+0.5)
  ax[1] <- a0(mx[1], sex[1])
  qx <- mx/(1+(1-ax)*mx)
  qx[m] <- ifelse(is.na(qx[m]), NA, 1)
  ax[m] <- ifelse(mx[m]==0, 0, 1/mx[m])
  px <- 1 - qx
  px[is.nan(px)] <- 0
  lx <- c(RADIX, RADIX*cumprod(px[1:OPENAGE]))
  dx <- lx*qx
  Lx <- lx-(1-ax)*dx
  Lx[m] <- lx[m]*ax[m]
  Tx <- rev(cumsum(rev(Lx)))
  ex <- Tx/lx
  avg.ex <- weighted.mean(ex, wx)
  avg.ex}

# calculating the average life disparity after disease diagnosis
avged <- function(mx, wx, sex){
  m <- length(mx)
```

```
  OPENAGE <- m - 1
  RADIX <- 1
  ax <- ifelse(mx==0, 0, mx*0+0.5)
  ax[1] <- a0(mx[1], sex[1])
  qx <- mx/(1+(1-ax)*mx)
  qx[m] <- ifelse(is.na(qx[m]), NA, 1)
  ax[m] <- ifelse(mx[m]==0, 0, 1/mx[m])
  px <- 1 - qx
  px[is.nan(px)] <- 0
  lx <- c(RADIX, RADIX*cumprod(px[1:OPENAGE]))
  dx <- lx*qx
  Lx <- lx-(1-ax)*dx
  Lx[m] <- lx[m]*ax[m]
  Tx <- rev(cumsum(rev(Lx)))
  ex <- Tx/lx
  dx.lx <- c(dx[-m]*(ex[-m]+ax[-m]*(ex[-1]-ex[-m])), dx[m]*ex[m])
  ed <- rev(cumsum(rev(dx.lx)))/lx
  avg.ed <- weighted.mean(ed, wx)
  avg.ed}

# calculating the average lifetable entropy after disease diagnosis
avghx <- function(mx, wx, sex){
  m <- length(mx)
  OPENAGE <- m - 1
  RADIX <- 1
  ax <- ifelse(mx==0, 0, mx*0+0.5)
  ax[1] <- a0(mx[1], sex[1])
  qx <- mx/(1+(1-ax)*mx)
  qx[m] <- ifelse(is.na(qx[m]), NA, 1)
  ax[m] <- ifelse(mx[m]==0, 0, 1/mx[m])
  px <- 1 - qx
  px[is.nan(px)] <- 0
  lx <- c(RADIX, RADIX*cumprod(px[1:OPENAGE]))
  dx <- lx*qx
  Lx <- lx-(1-ax)*dx
  Lx[m] <- lx[m]*ax[m]
  Tx <- rev(cumsum(rev(Lx)))
  ex <- Tx/lx
  dx.lx <- c(dx[-m]*(ex[-m]+ax[-m]*(ex[-1]-ex[-m])), dx[m]*ex[m])
  ed <- rev(cumsum(rev(dx.lx)))/lx
  Hx <- ed/ex
  avg.Hx <- weighted.mean(Hx, wx)
  avg.Hx}

# calculating threshold ages, which will be further used to separate the average life
disparity and average lifetable entropy into early and late components. We used the data
for people diagnosed with mental disorders as an example: mr = mortality rates, sex =
men/women, w = weight, i.e., the number of cases diagnosed at each age of onset
age <- 0:99
perturbation.ed <- horiuchi(func = avged, pars1 = diseased$mr, pars2 = diseased$mr*0.99,
                            N = 35, sex=diseased$sex[1], wx=diseased$w)
# calculating the threshold age for the average life disparity after diagnosis
f <- approxfun(age, perturbation.ed[(age+1)], method = "linear", rule = 2)
a.ed <- round(uniroot(function(x) f(x)-0, c(35,age[length(age)]))$root)

# calculating the threshold age for the average lifetable entropy after diagnosis
perturbation.hx <- horiuchi(func = avghx, pars1 = diseased$mr, pars2 = diseased$mr*0.99,
                            N = 35, sex=diseased$sex[1], wx=diseased$w)
f <- approxfun(age, perturbation.hx[(age+1)], method = "linear", rule = 2)
a.hx <- round(uniroot(function(x) f(x)-0, c(35,age[length(age)]))$root)

### Results for diagnosed people
diseased.results <- Metrics(diseased$age, diseased$mr, diseased$w, diseased$sex)
# obtaining the life disparity and the number of diagnosed cases at each age of onset
diseased.avged <- diseased.results[c("age", "ed", "wx")]
# separating the early and late average life disparity using the threshold age
diseased.avged$cutpoint <- c(rep("before", a.ed+1), rep("after", length(age)-(a.ed+1)))
# calculating the early and late average life disparity after disease diagnosis
avged.before <- subset(diseased.avged, diseased.avged$cutpoint == "before")
```

```
avged.after <- subset(diseased.avged, diseased.avged$cutpoint == "after")
avged.early <- weighted.mean(avged.before$ed, avged.before$wx)
avged.late <- weighted.mean(avged.after$ed, avged.after$wx)

# obtaining the lifetable entropy and the number of diagnosed cases at each age of onset
diseased.avghx <- diseased.results[c("age", "Hx", "wx")]
# separating the early and late average lifetable entropy using the threshold age
diseased.avghx$cutpoint <- c(rep("before", a.hx+1), rep("after", length(age)-(a.hx+1)))
# calculating the early and late average lifetable entropy after disease diagnosis
avghx.before <- subset(diseased.avghx, diseased.avghx$cutpoint == "before")
avghx.after <- subset(diseased.avghx, diseased.avghx$cutpoint == "after")
avghx.early <- weighted.mean(avghx.before$Hx, avghx.before$wx)
avghx.late <- weighted.mean(avghx.after$Hx, avghx.after$wx)
```
