# Supplementary material for: Lifespan variation among people with a given disease or condition
Source: PLoS One. 2023 Sep 1;18(9):e0290962. doi: 10.1371/journal.pone.0290962 (PMC10473533; doi:10.1371/journal.pone.0290962)
Supplement: Attachment — Submitted filename: Zheng_LifespanVariation_letter.pdf [file pone.0290962.s004.pdf]

Dear Dr Acharya,

Many thanks for allowing us the opportunity to revise and resubmit this manuscript. The reviewers have provided us with many constructive suggestions. We have addressed each reviewer's point below. Underlined represents additions to the original manuscript, while strikethrough texts represent deletions. Two versions of the revised manuscript have been uploaded (a ‘clean’ version, and one with track changes showing corrections). The page numbers refer to the version with track changes.

Editorial comments

We have changed the style, funding statement and ethics statement to meet the requirements. The funding statement now reads: “This work was supported by a Lundbeck Foundation Fellowship to Oleguer Plana-Ripoll (R345-2020-1588). Oleguer Plana-Ripoll has also received funding from Independent Research Fund Denmark (grants 1030-00085B and 2066-00009B). The funders of the study had no role in developing the methodology, study design, data collection, data analysis, data interpretation, or writing of the report. There was no additional external funding received for this study.”

Reviewer #1

This generally well-written article focuses on a method to estimate lifespan variation among people with a prevalent disease. While current demographic work has developed ways of measuring lifespan variation, combining these demographic ideas with the epidemiologic ones of disease prevalence has not been done before. In summary, this is a very well done combination of demographic and epidemiologic concepts. The methods seem robust, and the authors focus on a case study of Danish women with mental disorders. I have a few specific comments below:

Major comments:

1. Introduction: the introduction is well framed and outlines very well the justification for this study. A few comments: (a) “In the field of epidemiology, mortality metrics are fundamental for decision-making and resource distribution in the healthcare sector. “ The mission of epidemiology goes way beyond the healthcare sector, and I would argue that’s the sector where epidemiology hast the fewest to contribute (as opposed to social services planning, public health services planning, urban planning, etc.; in fact, line 70 mentions policymakers!); (b) I think a definition of life expectancy would be useful for a generalist journal such as plos one. This is especially important since the second paragraph mentions “a correct interpretation of the measure of life expectancy” but this interpretation is not provided.

RESPONSE: Thanks for your comments. We have made the following revisions:

“In the field of epidemiology, mortality metrics are fundamental for decision-making ~~and~~, resource distribution in the healthcare sector and designation of policies related to healthcare planning and social development.” (page 3, lines 59-61).

“An alternative and more intuitive mortality metric is life expectancy, which can be interpreted as the average number of years a synthetic cohort of newborns (or individuals at a specific age) are expected to live if they were to experience all age-specific mortality rates in that year.” (page 3, lines 63-65)

2. Metrics, L123: the authors mention that limitations to prior methods include “using a fixed age of onset” but it isn’t clear to this reviewer (and maybe to other readers) what this limitation is about.

RESPONSE: Previous studies have estimated life expectancy at one particular age, e.g. age 15 years. This is equivalent to assuming that everyone with the condition was diagnosed at that specific age. We have now added this information to the manuscript: “For instance, women and men with any type of mental disorder had 12 and 16 years of reduced life expectancy [7], and those with type 1 diabetes reported an estimated reduction of 10-12 years [5,8]. These studies, however, estimated remaining life expectancy at a fixed age (15 years for mental disorders and 20 years for type 1 diabetes [5,10]), which is equivalent to assuming that all cases were diagnosed at that age.” (page 7, lines 141-143)

3. Metrics, average LE after disease diagnosis: if I am understanding equation 2 right, this would be similar to estimating an age-adjusted/standardized (direct) mortality rate, but where we would be weighting the residual ex by a fixed distribution of ages at onset (instead of by the actual distribution of ages at onset). Is this right? If it isn’t, it may be good to think how to reword this.

RESPONSE: Yes, the average life expectancy after diagnosis could be conceptualized as a standardization of remaining life expectancy where the weights w(x) are taken from one of the groups but applied to both of them. We have rephrased this part to make it clearer: “With this method, it is possible to estimate the average life expectancy after diagnosis for those with a specific disease or condition, and an estimate can also be obtained for an age-matched comparison group using the same weights. It is important to use the same weights for the two groups so that the comparison is between groups of the same ages; thus, this average estimate can be conceptualized as a type of standardization.” (page 7, lines 162-163).

4. Metrics, early and late disparity: I suggest including an interpretation of e^dagger_0 and _1 in this section to help the reader understand how each one is interpreted differently.

RESPONSE: We have added the interpretations for the two metrics, which may help readers better understand what they mean: "where $\overline{e_0^\dagger}$ and $\overline{e_1^\dagger}$ are the average early life disparity and late life disparity after diseases diagnosis, respectively, and could be used to measure the lifespan variation before and after the threshold age $a^\dagger$ for people diagnosed with a given disease or condition" (page 9, lines 203-205)

5. Results, figures: the two figures are fantastic, and their labelling helps the reader. The only thing I'd add is an indication that the weight is fixed (footnote?)

RESPONSE: Thank you for the positive comment. We added the following indication in the figures: "The weights used to estimate average life expectancy and life disparity were the same for the two groups, namely, the number of diagnosed cases at each age."

6. Early vs late (results and discussion): if I am understanding things right, the authors find that Danish women with a diagnosed mental disorder live on average 7.3 fewer years than women without a diagnosis. They also find that life disparity is wider in general, but this is mostly driven by a wider life disparity before the threshold age vs a narrower one after the threshold age. However, for lifetable entropy women with a diagnosis have a wider variation both before and after the threshold age. The authors attribute this to higher overall mortality rates at any age for women with a diagnosis. Before the threshold age, this would increase lifespan variation (as higher mortalities before this age increase variation; see reference 21); however, after the threshold age, this would decrease lifespan variation. However, with entropy, as it takes into consideration ex, this phenomenon does not occur as it is more of a relative measure. Is this right? If it isn't, it may be good to think how to reword this.

RESPONSE: Yes, this is correct. We have added the following explanatory sentences to the manuscript: "As lifetable entropy reflects the relative variation in the length of life compared to life expectancy at birth [34], it may present contrasting dynamics compared with life disparity [35]. For instance, higher mortality rates at old ages among diagnosed individuals would reduce the life disparity; however, as it also decreases the corresponding life expectancy, it may increase the lifetable entropy (i.e., relative variation)." (page 13, lines 303-306).

7. Immortal time bias: the authors don't discuss this type of bias associated with diagnosis of conditions and I am wondering if they could think through and discuss its implications. My intuition is that since both ex and e^dagger_x are conditional on survival to a given age this wouldn't be an issue. The same would apply to H_x since it just relies on ex and e^dagger_x, but I wanted to make sure the authors have considered this, as metrics "following a diagnosis" tend to be a classical example of immortal time bias.

RESPONSE: This is a very good point and one of the strengths of these methods. We have added the following sentence to the discussion: "Additionally, individuals are followed from diagnosis, which means the estimates are conditioned on survival to that given age; thus, our method avoids any type of immortal time bias" (page 12, lines 292-293)

Minor comments:

1. Abstract: the first sentence speaks about individuals, but I'd say that life expectancy is useful to compare longevity across populations, not so much individuals. In the second sentence, the authors say that "The variation in lifespan […] has not been estimated", but I think it's missing something about the variation in lifespan IN people with a given condition. Last, the sentence "Unlike the average life disparity, for both early and late average lifetable entropy, women with mental disorders experienced larger estimates than those from the general population. " may benefit from some rewording to clarify (estimates of what?). For example "Unlike the average life disparity, both early and late average lifetable entropy was higher for women with mental disorders compared to the general population".

RESPONSE: We agree and have made changes accordingly: "life expectancy is also commonly used to investigate excess mortality among a group of individuals diagnosed with specific diseases or conditions"; "the variation in lifespan – a measure commonly used in the field of demography – has not been estimated for people with a specific condition"; and "Unlike the average life disparity, ~~for~~ both early and late average lifetable entropy~~,~~ were higher for women with mental disorders ~~experienced larger estimates than those from~~ compared to the general population" (page 2).

2. Data: I don't see any description of how the Danish VRS is linked with the Danish Psychiatric Central Research Register.

RESPONSE: We have now added a description regarding the linkage of the two data sources: "The two registers were linked on an individual level using a unique personal identification number, which was assigned to all legal residents at birth or immigration to Denmark [28]." (page 5, lines 110-112)

3. Metrics, L118: This sentence is missing something at the end ("among those diagnosed" with…?).

RESPONSE: We have changed the sentence to "…among those diagnosed with a particular condition" (page 6, line 137).

Reviewer #2

1. The paper presents some very interesting measures of life expectancy variation among people with a given disease, averaged over age at diagnosis. As such, the idea can be welcomed as it potentially provides a new way of looking at inequalities between people with a given diagnosis. Implicitly, inequality in life expectancy also suggests how quality of life may differ between the healthy and the sick: do the sick suffer both a shorter life expectancy and a longer period of severe disability before death, or at least could the period of severe disability before death be assumed not to be protracted? In fact, the rich discussion on the topic of mortality and morbidity compression/expansion started (with [1]) with this very important question in mind: if we extend life expectancy, do we also extend the period of severe disability before death? Apart from the individual quality of life before death, the question is also of public health concern: prolonged periods of disability imply higher health care costs as life expectancy increases. The answer to this question at the population level is negative: life expectancy increases, but disability and mortality are compressed into shorter periods of cohort life. There is value in extending this discussion to disease-specific contexts. However, this brings me to the major problem of the paper, which should be corrected before publication: the authors do not relate their metrics to the traditional issues of mortality/morbidity compression/expansion. One, perhaps more intuitive, way of doing this might be (in addition to a refined comparison and discussion of the variation in life expectancy between people with different health conditions) to look at the temporal change in the metrics produced.

RESPONSE: Thanks for your comments – this is a very important issue. First, for this study, we used data for the period 1995-2018, and we considered the possibility of looking at temporal changes in the metrics produced over this period. However, we then realized that this would be a huge study in which we should have to describe the metrics (as we do now) and then look at temporal changes. Instead, we have decided to focus on this first introductory study where we just describe the new metric used. We plan to look further into the topic by considering different types of mental disorders (e.g. depression, anxiety, schizophrenia, etc.) for both men and women and explore potential changes over time. We believe this would provide a comprehensive overview of lifespan variation among those suffering from mental disorders, taking into account that specific mental disorders are different from each other. Second, we would like to emphasize that we are not looking at morbidity or quality of life (besides being diagnosed with a mental disorder, which might be potentially disabling). However, we agree the mortality/morbidity compression/expansion is important. We have added the following parts to the manuscript: "It is important to notice that these formulas are based on mortality rates in a given period, but we are ignoring the time component in this study as it would unnecessarily complicate the descriptions. <u>In future studies, it is important to consider potential time trends in such metrics</u>" (page 7, line 165) and "<u>This divergence reveals the disagreement when using different metrics to measure the variation in lifespan, which again underlines the crucial influence of the age-at-death distribution. This issue will</u>

be particularly important when investigating these metrics across periods as mortality/morbidity compression or expansion, particularly the mortality shift at older ages would play an important role in affecting the longitudinal trend in lifespan variation [36,37], which will also be applied to the average lifespan variation measures" (page 13, lines 310-316).

On a more technical side:

2. Mortality shifts (in either direction) can lead to false (de)compression effects if life expectancy change metrics are calculated for fixed ages [2] - this seems to me to be an issue for the age-averaged metrics proposed in the paper as well. Consider a scenario where the mortality of those with a disease is shifted to younger ages compared to the main population. How would your metrics react (ideally, there should be no change in life expectancy variation)?

To illustrate, I run a simple scenario that mimics your parameters using the 2014 Australian life table: I compare the original life table with one where the mortality curve is shifted 7 years younger along the age axis. Life expectancy at age 39 is closest to your average life expectancy of 44.9 in the general population; it is 7 years shorter (because of the shift) in the worse mortality case. Looking at e-dagger at 39, one would find that it shrinks by 0.3 years from 9.2 in the original to 8.9 in the 'shifted' scenario. Thus, a pure shift induces a false compression for those 'diagnosed' at age 39. This spurious effect is opposite in sign to the one you report, but comparable in magnitude! Thus, after somehow correcting for the shifting effect, one would get an effect almost twice as large as the one reported in the paper. /For entropy, the effect of the shift is more complex.

RESPONSE: This is an interesting point, and thanks for these additional analyses. We have added the following sentence to the discussion: "mortality shifts could potentially lead to false (de)compression effects as life expectancy is measured at fixed ages [37]. Thus, in the scenario in which those with a given disease experience reduced life expectancies, the differences in lifespan variation would be expected to be larger than the observed ones" (pages 13-14, lines 325-328).

3. Entropy is a different story from e_dagger. Unlike e_dagger, which more explicitly reflects variation in age at death, H is useful as a tool to roughly infer the effects of proportional mortality reduction/increase on life expectancy [3]. This is an interesting research perspective in itself, perhaps even more so in the context of reducing cause-(diagnosis-) specific mortality. I suggest you address this aspect in your paper to better link it to the existing literature on H.

RESPONSE: We have expanded the definition of entropy in the methods section to highlight its importance and its difference from other lifespan variation metrics. Additionally, we have revised the discussion section by linking it to the existing literature: "As lifetable entropy reflects the relative variation in the length of life compared to life expectancy at birth [34], it

may present contrasting dynamics compared with life disparity [35]. For instance, higher mortality rates at old ages among diagnosed individuals would reduce the life disparity; however, as it would also decrease the corresponding life expectancy, it may increase the lifetable entropy (i.e., relative variation). This divergence reveals the disagreement when using different metrics to measure the variation in lifespan, which again underlines the crucial influence of the age-at-death distribution. This issue will be particularly important when investigating these metrics across periods as mortality/morbidity compression or expansion, particularly the mortality shift at older ages would play an important role in affecting the longitudinal trend in lifespan variation [36,37], which will also be applied to the average lifespan variation measures." (page 13, lines 303-316)

4. After addressing the points above, your discussion section may also need to be redesigned. Overall, the idea is great indeed, but the presentation needs a major improvement in order to fully realize it (the idea).

RESPONSE: We have revised the discussion section considerably by adding additional information on entropy (page 13, lines 303-316) and an entire new paragraph with a few considerations to take into account (pages 13-14, lines 318-330).

5. Possible typos:

[1] In eq. (1), shouldn't you use another index (say i for individuals), not age x?

RESPONSE: We agree it is more clear to based the index on individuals instead of age and have changed the formula to: $\bar{e} = \frac{\sum_{i=1}^{n} e(x_i)}{n}$

[2] Eqs (4), (5) – shouldn't they have different denominators n0 and n1, not the total n?

RESPONSE: Apologies for this mistake – it is now fixed.

Reviewer #3

The manuscript proposes to estimate lifespan variability of individuals that have been diagnosed mental disorders. The motivation is that while it is common practice to estimate average lifespan (via life expectancy) of individuals with a specific disease, it can be useful to measure also inequality of lifespans. Then the new metric is applied to Danish register data.

1. The paper is overall well written, and the idea of using a measure of lifespan disparity is interesting. However I have one concern about the way this new metric is presented. If authors' intention is

proposing a new measure, then I think they should devote more space to mathematical/statistical properties of it. What is the uncertainty related to this measure? How much is sensitive to outliers or to values at higher ages where death rates - and thus life expectancies, are more volatile? Some diagnostic and discussion of this measure should be provided.

I understand that this would mean a drastic transformation of the paper but at the present time, the manuscript lies in between a methodological paper and an applied one. I think this ambiguity should be fixed.

RESPONSE: Thanks for your comments. We have built the definition of average lifespan variation after diagnosis based on previous studies describing the average life expectancy after diagnosis. We suggest estimating uncertainty and confidence intervals around this estimate using the same approach, namely through non-parametric bootstrap. We have now added the following sentence to the discussion section: "Because the aim of this paper was to discuss metrics to estimate lifespan variation among those with a given disease, we have not calculated a measure of uncertainty. However, standard errors and confidence intervals may be obtained via non-parametric bootstrap" (page 14, lines 328-330). We have included more details of the mathematical properties of the observed metrics in the methods section. Additionally, we have mentioned the limitations of such metrics in relation to outliers and uncertain mortality rates in the discussion section: "the proposed measures are based on mortality rates, which might not be accurate at very old ages (e.g., having extreme values) in some countries, particularly developing countries, which means that smoothing techniques for adjusting the observed mortality rates might be necessary. However, the ages in which there are more cases of mental disorders will have a higher weight on the average measures we propose; thus, extreme values might have a lower impact here than on standard non-averaged mortality metrics" (page 13, lines 318-322).

MINOR:

2. Table 1 refers to a "cohort" of individuals. It is not entirely clear to me what do you mean by "cohort" here. To my knowledge a cohort is a set of individuals that underwent a specific event (the "origin" event) in the same year. It is clear that the starting event is a diagnosed mental disorder, but it is not clear what is the common year. Maybe you could explain a little more about this.

RESPONSE: We have used the definition of cohort as in many epidemiological designs as a group that is followed from one point (diagnosis of a mental disorder) onwards – compared to case-control studies that would look retrospectively from a given point in time. However, to avoid any confusion, we have deleted the word cohort from the manuscript.

3. Figures come at a very low quality. I understand that in some cases is the editorial manager to transform high quality images into blurred figures, however I recommend authors to ensure that figures have a good definition

RESPONSE: We have provided the figures in good quality – we hope the final published figures will be the accurate ones.

4. I applaud to authors' choice to share the R code, but at the same time I suggest to do that in a way that makes it more readable, for instance using comments to explain what a specific chunk is intended for

RESPONSE: We have added comments in the revised R file to make the codes more readable to the readers.
